# Xenogeneic cell therapy provides a novel potential therapeutic option for cancers by restoring tissue function, repairing cancer wound and reviving anti-tumor immune responses

**DOI:** 10.1186/s12935-018-0501-7

**Published:** 2018-01-16

**Authors:** Chi-Ping Huang, Chi-Cheng Chen, Chih-Rong Shyr

**Affiliations:** 1 Department of Urology, Graduate Institute of Clinical Medical Science, China Medical University and Hospital, 40454, Taichung, Taiwan; 2 Department of Urology, Taichung Tzu Chi Hospital, Buddhist Tzu Chi Medical Foundation, Taichung 404, Taiwan; 3 Sex Hormone Research Center, Department of Laboratory Science and Biotechnology, China Medical University and Hospital, 6 Hsiuh-Shih Rd, Taichung, 40454 Taiwan

**Keywords:** Xenogeneic cell, Cancer, Wound, Chemotherapy, Targeted therapy, Immunotherapy, Tumor microenvironment, Immune, Xenotransplantation, Anti-tumor, Immunity

## Abstract

Conventional cancer treatments such as surgery, radiotherapy, chemotherapy and targeted therapy, not only destruct tumors, but also injure the normal tissues, resulting in limited efficacy. Recent advances in cancer therapy have aimed at changing the host milieu of cancer against its development and progression by targeting tumor microenvironment and host immune system to eradicate tumors. To the host body, tumors arise in tissues. They impair the normal healthy tissue physiological function, become chronically inflamed and develop non-healing or overhealing wounds as well as drive immuno-suppressive activity to escape immunity attack. Therefore, the rational therapeutic strategies for cancers should treat both the tumors and the host body for the best efficacy to turn the deadly malignant disease to a manageable one. Xenogeneic cell therapy (i.e. cellular xenotransplantation) using cells from non-human source animals such as pigs has shown promising results in animal studies and clinical xenotransplantation in restoring lost tissue physiological function and repairing the wound. However, the major hurdle of xenogeneic cell therapy is the host immunological barriers that are induced by transplanted xenogeneic cells to reject xenografts. Possibly, the immunological barriers of xenogeneic cells could be used as immunological boosters to activate the host immune system. Here, we hypothesized that because of the biological properties of xenogeneic cells to the recipient humans, the transplantation of xenogeneic cells (i.e. cellular xenotransplantation) into cancer patients’ organs of the same origin with developed tumors may restore the impaired function of organs, repair the wound, reduce chronic inflammation and revive the anti-tumor immunity to achieve beneficial outcome for patients.

## Background

Current standard treatments such as surgery, radiotherapy and chemotherapy, although effective, come with collateral damages of injuring healthy tissue and causing a number of complications and adverse effects. Furthermore, the outcome is discouraging for most advanced cancer patients [1]. Tumors arise in normal tissues, and therefore, conceptually cancers can be considered a pathological imbalance of tissue-cell societies, where normal tissue homeostasis and architecture could inhibit progression of cancer, but cancer cells are able to abnormally expand and recruit normal cells, such as fibroblasts, endothelial cells and inflammatory cells to take advantages of the host’s physiological functions to form a tumor, which comprise a tumor favorable microenvironment [2, 3]. Since the microenvironment is shifted into the situation that facilitates tumor development progression and metastatic dissemination with signals or tissue architectures promoting cell proliferation and survival and stimulating cell migration and invasion, novel therapies are proposed to targets the tumor microenvironment. For example, inhibition of VEGF signaling with anti-angiogenic agents is used to inhibit neo-angiogenesis to repress tumor vasculature in tumor microenvironment and stop tumor growth and survival [4]. This type of therapy has transformed therapeutic concept that in addition to directly remove cancers with surgery, cytotoxic and targeted anti-tumor agents, targeting tumor microenvironments and host tissues could be another effective approaches to treat cancer.

The advances of immunotherapy, either immune-checkpoint blockade monoclonal antibody therapy or adoptive cellular therapy have make big breakthrough on cancer treatment with unprecedented responses for advanced-stage cancer patients who fails conventional chemotherapy and targeted therapy [5]. This approach is similar to the anti-tumor microenvironment therapy because, instead of focusing on the strategies that directly eliminate cancer cells, their strategy is to target the changes in the host body, triggered by tumors. The responses of the body to a cancer also parallel to the body’s wound healing responses, and therefore, it is considered cancer as a wound that don’t heal or an overhealing wound [6, 7]. Thus, to treat cancers, this complex network of host responses offers targets for prevention and treatment of malignant disease.

Xenotransplantation is defined in FDA Guidance as any procedure that involves the transplantation, implantation or infusion into a human recipient of either (a) live cells, tissues, or organs from a nonhuman animal source, or (b) human body fluids, cells, tissues or organs that have had ex vivo contact with live nonhuman animal cells, tissues or organs. The use of animal organs in humans has been long been tested: a baboon heart was transplanted into a newborn infant, Baby Fae, who had hypoplastic left heart syndrome and lived 20 days after heart surgery [8] and a baboon liver was transplanted to a patient with hepatic failure [9]. In addition to the replacement of the heart, lungs, liver, and kidneys from non-human animals to human, xenotransplantation is also being developed to use xenogeneic cells such as porcine islet cell for diabetes, porcine dopaminergic neurons for Parkinson’s disease and porcine hepatocytes for liver failure, which all demonstrate cross-species physiologic activity and metabolic regulation in host human tissues [10–12]. With the advent of genetic engineering, genomic editing and cloning technologies, the pathobiological barriers to successful porcine organ xenotransplantation might be resolved by transplanting organs from genetically engineered pigs such as α1,3-galactosyltransferase gene-knockout (GTKO) pigs to delete xenoantigens on pig organs for protecting them from the human immune responses [13, 14]. The recently developed [clustered regularly interspaced short palindromic repeat (CRISPR)/CRISPR-associated 9 (Cas9)] genomic editing technology has also been applied to generate major histocompatibility complex (MHC) class I null pigs [15] and α1,3-galactosyltransferase and cytidine monophosphate-N-acetylneuraminic acid hydroxylase gene double-deficient pigs [16] to prevent rejection in xenotransplantation.

The most profound obstacle to xenotransplantation is the immunological rejection of the organ, tissue or cell grafts [17]. Graft rejection events include, the antibody-mediated processes: hyperacute rejection (HAR) and acute humoral xenograft rejection (AHXR), which rapidly attack vascularized organs by host antibody and immune cell binding to the vascular endothelium of the xenograft, leading to its destruction and immediate loss of function [11, 17]. Anyhow, if HAR and AHXR are prevented, the xenograft is subjected to cellular immune responses through pathways that are similar to the rejection pathways of allografts mediated by histocompatibility determinants and other cell surface components, involving helper and killer T cells and others to cause graft failure [11, 17]. HAR causes xenograft immediate loss of the function of the transplant with diffuse interstitial hemorrhage, edema and thrombosis of small vessels due to the binding of human humoral antibodies to a sugar epitope, the Gal epitope (galactose-a-1,3-galactose), which is present on the vascular endothelium [11, 17]. AHXR on vascularized xenografts several days to weeks after transplantation can be induced by very low levels of α1,3Gal-specific natural antibodies or xeno reactive antibodies specific for non-α1,3Gal antigens and complement activation or complement-independent mechanisms may also contribute to the pathogenesis of AHXR [11, 17]. Because of lack of vascularization for cells, recent clinical xenotransplantation trials are conducted, not using organs, but xenogeneic cells because HAR and AHXR vascular rejection do not occur [10]. Therefore, xenogeneic cell therapy like islet xenotransplantation using porcine islets would be more close to the clinical reality than using the whole organ.

## Presentation of the hypothesis

**Fig. 1:**
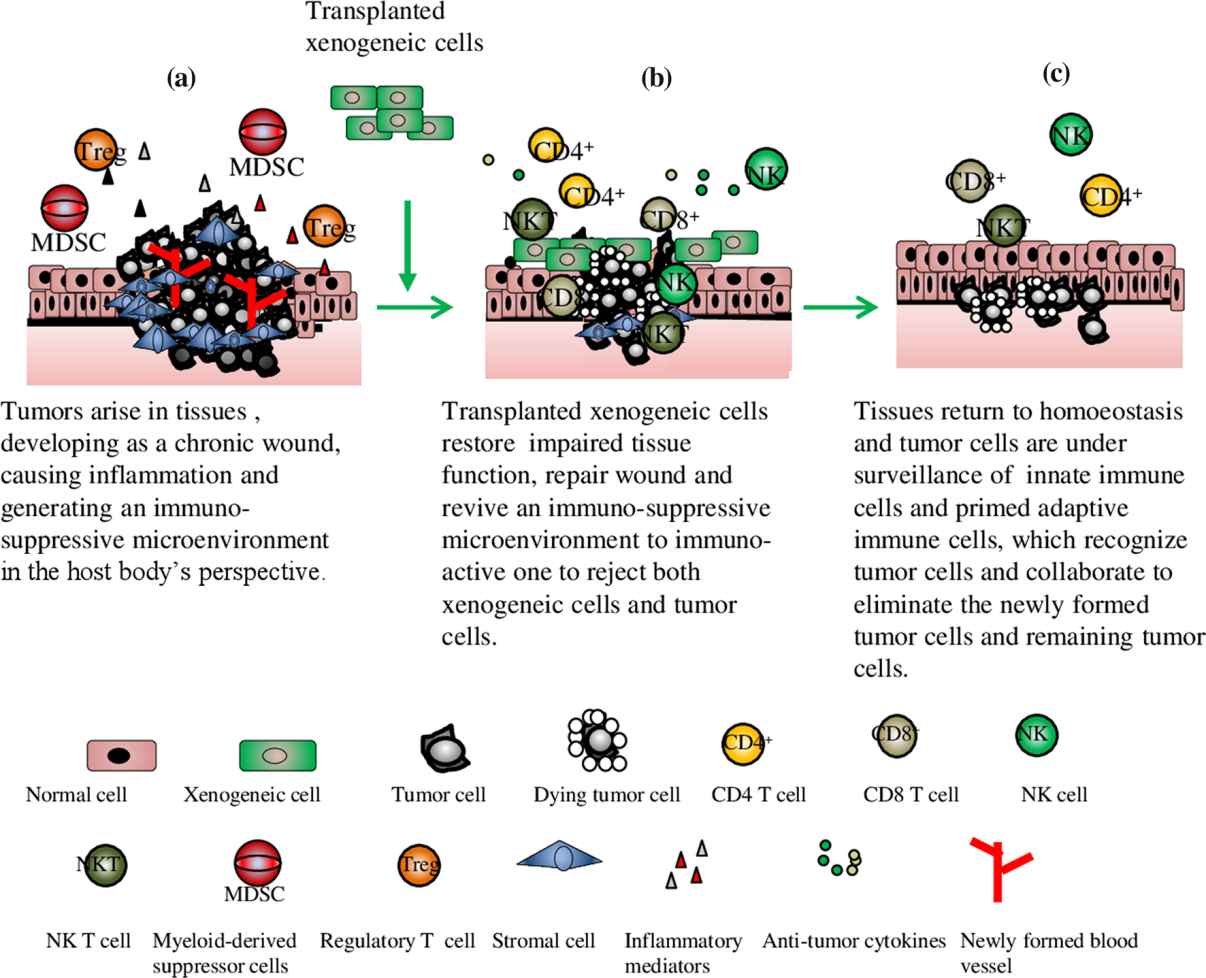
Proposed therapeutic actions of xenogeneic cells on cancers. **a** Progressive tumors in tissues impair tissue function, generate non-healing or overhealing wounds with neo-angiogenesis, induce chronic inflammation along with inflammatory mediators and are often infiltrated by myeloid-derived suppressor cells and regulatory T cells to create an immuno-suppressive microenvironment with the help of T cell checkpoint inhibition. **b** Transplanted xenogeneic cells of the same tissue origin could restore impaired tissue function, promote wound healing to reduce inflammation and induce immunological rejection responses to revive the immuno-suppressive microenvironment to immno-active one for rejecting both xenogeneic cells and tumor cells by the collaboration of CD4+ T helper cells cytotoxic CD8+ T cells, NK, and NK T cells as well as a set of anti-tumors cytokines, resulting in tumor regression. **c** Tissues return homeostasis with resolution of inflammation and healed wound. Tumors shrink and the innate immune cells and primed adaptive immune cells survey the tissues to contain the tumor growth by recognizing and eliminating newly formed tumor cells and remaining tumor cells

Based on the pathogenesis of cancers and the therapeutic values of xenogeneic cell therapy (i.e. cellular xenotransplantation), we hypothesized that the transplantation of xenogeneic cells into organs of the same origin (ex. porcine hepatocytes into livers of hepatocellular carcinoma patients), which are inflicted by cancers may restore the impaired function of organs damaged by overgrowing cancer cells, repair the wound and reduce chronic inflammation caused by aberrant cancer cells and revive the anti-tumor immune responses suppressed by evading cancer cells. Since the xenogeneic cells of different organs from higher mammals such as bovine or porcine sources have share similar functions as human, possess the wound healing ability and carry non-human antigens that activate host cellular rejection mechanism. The use of xenogeneic cells may improve the therapeutic outcome of cancer patients in combination with anticancer drugs to increase life quality and extend the survival of patients with malignant diseases. The overall propose actions of xenogeneic cells on cancer are illustrated in Fig. 1.

## Test of the hypothesis

The clinical xenotransplantation has been directed to the use of xenogeneic cells which don’t have vasculatures and are not subjected to HAR and AHXR rejection [11, 17]. Porcine islets were transplanted into diabetic nonhuman primates and demonstrated that porcine islets restore glucose control and prolonged survival [18, 19]. Porcine fetal neural cells have been grafted unilaterally into Parkinson’s disease and Huntington’s disease patients and some clinical improvement has been observed [20]. These clinical cellular xenotransplantation studies demonstrated that xenogeneic cell therapy is a promising approach to a variety of human diseases and disorders characterized by cellular dysfunction or cell death to restore dysfunction of human receipt’s organs. That is because the source animals like pigs have similar physical or physiological characteristics comparable to human and therefore, there is cross-species physiologic activity and metabolic regulation of xenogeneic transplanted cells occurring in host tissues. As cancers develop in tissues, because the rise and spread of cancer cells, they could cause irreversible and terminal tissue and organ failure and when cancer affects a vital organ such as brain, lung, liver and kidney, the organ function is impaired and progresses to failure, resulting in death. For example, tumors in liver could induce symptoms of advancing cirrhosis, impair liver function and in severer form, result in liver failure, causing death [21], so liver transplantation is the best treatment option for patients with early-stage tumor. Hence, transplantation of xenogeneic porcine hepatocytes into liver cancer patients could restore partial liver function.

As mentioned before, cancers develop because tissues are in injury and develop wounds where cell proliferation is enhanced by multiple growth factors and cytokines to help tissue regenerates like that cancer cells hijacking wound healing responses for their own benefits. Genetically and epigenetically altered cancer cells with proliferative potentials at the injury sites, assisted by inflammatory cells and growth/survival factors dominate the tissues to grow as a chronic wound fail to heal or an overhealing wound [6, 7, 22]. Furthermore, surgery, chemotherapeutic agents and radiation therapy all collaterally damage normal tissues to cause more inflammation and wound that could facilitate cancer progression and spread. Therefore therapeutic approaches that target wound healing and inflammation could provide another mechanism of action to control cancer growth, metastasis, and response to therapy. Xenogeneic cell therapy may provide such actions to promote wound healing and reduce cancer-promoting chronic inflammation once they are transplanted into tumor sites as porcine skin xenografts do on wounds.

Immunotherapy on cancer is based on the concept that the activation of the host immune system either by immune checkpoint inhibitor anti or genetically engineered T cells could eradicate tumors that escape from immune system. Both innate and adaptive immunity are involved in cancer immunosurveillance that particular innate and adaptive immune cell types such as T, B, and natural killer T (NKT) lymphocytes, NK cells, dendritic cells (DCs), effector molecules, and regulatory pathways are collectively functions to suppress tumor formation [23]. The cancer immunoediting process has been involved in the therapeutic effects of immunotherapies against cancer with immune checkpoint inhibitors such as CTLA-4, PD-L1, and PD-1 blockade, which target tumor escape mechanisms to reverse host immunosuppressive state. It is of great interest to design the therapeutic strategies that harness the power of immunity to eliminate transformed tumor cells by boosting anti-tumor immune responses. Recent advances in genomic sequencing and bioinformatics have delineated the anti-tumor immune responses to cancer with the understanding the nature of cancer neoantigens that are expressed exclusively in and on tumor cells, generated due to progressive mutational process that drives cancer evolution and these cancer neoantigens generate peptide epitopes presented by DC cells to induce T cell compartment that recognize their displays on major histocompatibility complexes on the surface of the malignant cells and reject transformed cancer cells [24]. Comparing the molecular and cellular mechanisms of the immune responses exerted by the host to reject transformed cancer cells or reject transplanted xenogeneic cells, there are many similarities. Both cells are considered non-self cells by the host body to be rejected from the body with innate and adaptive immunity [25, 26]. Without vasculature, anti-xeno antibodies in human sera don’t induce antibody response and lead to hyperacute rejection, but instead, T cells, NK cells and macrophages play a major role in rejecting transplanted xenogeneic cells [25]. These cellular immune responses on xenoantigens play a more important role in cellular xenotransplantation as in allografts, compared to solid organ xenotransplantation [27]. Xenogeneic T cell responses involve recipient T cell stimulation via xenoantigen peptide presentation (on recipient class II molecules) by recipient antigen presenting cells and are similar in strength and specificity to an allogeneic response with the T-cell receptor repertoire, accessory molecule interactions and cytokine production [28]. Xenogeneic T-cell-mediated rejection is suggested to involve cytotoxic T cell killing, helper T cell with IL-2 stimulation or CD4 T-cell stimulation to activate NK-mediated direct cellular killing [28]. And the T cell costimulation blockade immunosuppression regimen was used for the xenotransplantation of porcine islets into nonhuman primates by using anti-IL-2 receptor, and anti-CD154 antibodies [18, 19]. In addition to cellular rejection, transplanted xenogeneic cells are also the targets of host humoral immune responses such as instant blood-mediated inflammatory reaction on transplanted pig islets with the activation of platelets, the coagulation and the complement systems and leukocyte infiltration, resulting in islet loss [29].

Therefore, it is possible that the transplantation of xenogeneic tissue-specific cells into the specific tissues that are afflicted with tumors will induce cellular and humoral immune rejection to reject xenogeneic cells, which concomitantly revive anti-tumor immune responses to reject tumors.

Although recent immune check point inhibitors (anti-CTLA4 and anti-PD/PD-L1 antibodies) make a significant breakthrough in treating recurrent or metastatic cancers, the response rate is limited and some responsive patients still develop resistance and progress [30]. Compared to immune checkpoint inhibitors, our hypothesized xenogeneic cell therapy strategy would induce natural rejection immune responses including all aspects of innate and adaptive immunity [25], which may also collaterally revive multiple anti-tumor immune responses and achieve higher response rate and more durable cancer control. In contrast, immune check point inhibitors artificially target and block one specific immune inhibitory pathway, which may result in treatment unresponsive in patients with tumors lack of checkpoint molecule expression like PD-L1, and failure in patients who develop resistance [30]. Additionally, normal and functional xenogeneic cells are transplanted to cancerous organs, which may improve physiological functions of patients, but for immune checkpoint blockade, since immune inhibitory pathway is artificially and specifically blocked by antibodies, the dysregulated immunity could cause a wide variety of immunotherapy-related adverse events in patients ranging from mild to severe life-threatening [31].

Before clinical application, preclinical animal studies can be used to evaluate the efficacy and safety of xenogeneic cellular therapy on cancer to suggest the dose, route of administration and possible mechanisms of action and predict possible adverse events. Modelling cancer in mice has advanced to allow investigators can study complex process of cancer development and progression and test novel therapeutics with approaches such as grafting mice with tumor cell lines or explants, using chemical and viral carcinogens to induce tumors, and producing genetically engineered mice that develop tumors [32]. These murine cancer models can be used in the preclinical studies for evaluating the effects of xenogeneic cellular transplantation to determine the efficacy and pathobiological changes in tumors to investigate the fates and actions of transplanted xenogeneic cells on tumors.

## Implications of the hypothesis

If our hypothesis is proven correct, it will transform our cancer treatments, not just focusing on cancer killing, but also improving the host body ability to heal the cancer injury and wound. Cancers cause morbidity and mortality because they spread and can disrupt the functioning of normal vital organs such as lungs, livers, kidney, pancreases and brains. Xenogeneic cells, such porcine hepatocytes, pancreatic cells and neural cells pose the similar functions as human counter parts and have been shown functional in clinical and animal models to improve organ functions [33, 34]. If xenogeneic cells isolated from a particular organ are transplanted into the same organs with tumors in patients, we expect these xenogeneic cells will partially restore the physiologic functions disrupted by the cancers cells. The tissue architecture could also provide the scaffolds for the transplanted cells to grow and engraft until they are rejected by the immune responses. Furthermore, in clinical allo-transplantation for cancers, if the immune response in graft rejection would help boost anti-tumor immunity to contain tumors, the active immunity could have effects on tumor recurrence and progression. For example, liver transplantation is the treatment of choice for hepatocellular carcinoma, and the study found that lower recurrence-free survival was related to high dosage of the immunosuppressant cyclosporine administered during postoperative months 3–12 [35], suggesting graft rejection immunity would prevent tumor development and progression, which supports our hypothesis that the rejection to grafts could turn on anti-tumor responses.

Cancer is a major leading cause of death throughout the world and despite the extraordinary amount of effort and money expended on basic and clinical researches over the past several decades, it is still a formidable challenge to effectively eradicate or control advanced cancers with current best cancer therapies. If our hypothesis is proven correct, we turn the immunological hurdle disadvantage of xenogeneic cells into immune adjuvant advantage to boost the receipt’s host immune system to eradicate tumors. This new therapeutic strategy will transform our cancer treatments: not just focusing on cancer killing, but also improving the host body ability to heal the cancer injury and wound. Although further preclinical and clinical studies are needed to demonstrate the safety, efficacy and mechanism of action of xenogeneic cell therapy in which setting of cancers (early, advanced or metastatic cancers) for proving our hypothesis, we believe with this novel xenogeneic cell therapy modality for cancer treatment, cancer diseases could become a manageable disease, not a devastating one.

## Abbreviations

HAR: hyperacute rejection
AHXR: acute humoral xenograft rejection
NK: natural killer
PD-L1: programmed cell death protein ligand 1
DC: dendritic cell
